# Epigenetic silencing by the SMC5/6 complex mediates HIV-1 latency

**DOI:** 10.1038/s41564-022-01264-z

**Published:** 2022-11-14

**Authors:** Ishak D. Irwan, Hal P. Bogerd, Bryan R. Cullen

**Affiliations:** Department of Molecular Genetics and Microbiology, Duke University Medical Center, Durham, NC USA

**Keywords:** Restriction factors, HIV infections, Gene silencing, Viral reservoirs, Viral host response

## Abstract

After viral entry and reverse transcription, HIV-1 proviruses that fail to integrate are epigenetically silenced, but the underlying mechanism has remained unclear. Using a genome-wide CRISPR/Cas9 knockout screen, we identified the host SMC5/6 complex as essential for this epigenetic silencing. We show that SMC5/6 binds to and then SUMOylates unintegrated chromatinized HIV-1 DNA. Inhibition of SUMOylation, either by point mutagenesis of the SMC5/6 component NSMCE2—a SUMO E3 ligase—or using the SUMOylation inhibitor TAK-981, prevents epigenetic silencing, enables transcription from unintegrated HIV-1 DNA and rescues the replication of integrase-deficient HIV-1. Finally, we show that blocking SMC5/6 complex expression, or inhibiting its SUMOylation activity, suppresses the establishment of latent HIV-1 infections in both CD4+ T cell lines and primary human T cells. Collectively, our data show that the SMC5/6 complex plays a direct role in mediating the establishment of HIV-1 latency by epigenetically silencing integration-competent HIV-1 proviruses before integration.

## Abstract (web summary)

HIV-1 latency is enabled by SMC5/6-mediated epigenetic silencing of unintegrated viral genomes.

## Main

Integration of proviral DNA into the host cell genome is a defining feature of the retroviral life cycle that is essential for proviral transcription and replication^1,2^. Integrase (IN) inhibitors potently inhibit HIV-1 replication^3^. In the absence of functional IN, unintegrated HIV-1 proviruses accumulate repressive epigenetic marks, including trimethylation of lysine 9 on histone H3 (H3K9me3), and are depleted of activating marks, such as H3 acetylation (H3Ac)^4,5^. While the epigenetic silencing of transcription from unintegrated HIV-1 DNA probably represents a host defence against foreign DNA, the underlying mechanisms and cellular factors that mediate this effect remain incompletely defined^6,7^.

In murine leukaemia virus (MLV), a genomic screen identified components of the human silencing hub (HUSH) complex, as well as the DNA-binding protein NP220, as critical for unintegrated MLV DNA silencing^8^. However, subsequent work^9,10^ failed to detect any role for the HUSH complex or NP220 in silencing unintegrated HIV-1. More recently, a screen of 1,217 human genes found to be downregulated by the HIV-1 Vpr protein identified a component of the structural maintenance of chromosome (SMC) 5/6 complex, SMC5/6 complex localization factor 2 (SLF2), as critical for unintegrated HIV-1 DNA silencing. This screen also showed that six other components of the SMC5/6 complex, including SMC5 and 6 as well as the four SMC5/6 associated proteins non-structural maintenance of chromosomes element 1 through 4 (NSMCE1–4), but not the SMC5/6 associated factor SLF1, were also critical for the epigenetic silencing of unintegrated HIV-1 DNA^9^. Of note, the SMC5/6 complex was previously shown to be degraded by the hepatitis B virus (HBV) non-structural protein HBX and, in the absence of HBX, episomal HBV DNA is also epigenetically silenced^11,12^. Thus, the SMC5/6 complex not only participates in chromosomal replication, recombination and repair^13^ but can also silence invasive viral DNA. Here we sought to determine whether the SMC5/6 complex mediates the establishment of latent HIV-1 infections.

## Results

### A genomic screen for factors that silence HIV-1 proviruses

**Fig. 1:**
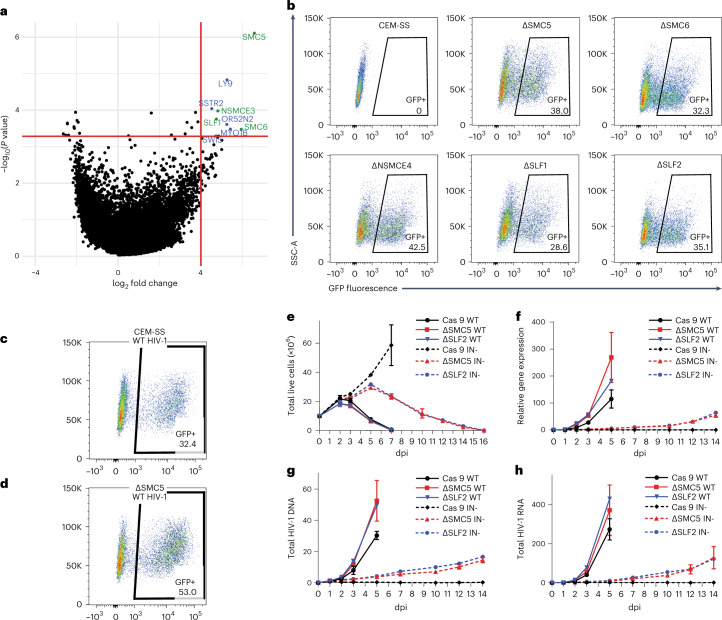
Screen identifies a role for SMC5/6 in silencing unintegrated HIV-1 DNA. **a**, Volcano plot of the mean fold change of sgRNAs specific for each gene and their false discovery rate (FDR)-corrected *P* values. Genes with fold change >16 and *P* value <0.0005 (delineated by the red lines) are labelled. Briefly, *P* values for individual sgRNAs were calculated using a negative binomial (NB) model, then sorted sgRNA *P* values were used to calculate FDR-corrected *P* values for individual genes using the robust rank aggregation (αRRA) algorithm in MAGeCK-VISPR^50,51^. Members of the SMC5/6 complex are in green (FDR-corrected *P* values: SMC5 = 7.8 × 10^−7^, SMC6 = 0.00033, NSMCE3 = 0.00010, SLF1 = 0.00017). **b**, Flow cytometry of WT CEM-SS cells and the indicated clonal knockout cell lines at 2 dpi with an IN− NL-GFPΔEnv reporter virus at an MOI of ~0.3. A representative experiment from 3 biological replicates is shown. **c**,**d**, Flow cytometry of WT (**c**) or ΔSMC5 CEM-SS (**d**) cells at 2 dpi with IN+ NL-GFPΔEnv reporter virus at an MOI of ~0.3. Representative experiments from 3 biological replicates are shown. **e**–**h**, Time course of the infection of the parental CEM-SS Cas9 cells, or the ∆SMC5 and ∆SLF2 CEM-SS clones with IN+ or IN− NL-NLuc. **e**, Live cells. **f**, Virally encoded NLuc expression. **g**, Total HIV-1 DNA**. h**, Total HIV-1 RNA expression quantified at the indicated dpi. All IN+ HIV-1-infected cultures died from viral cytopathicity by 7 dpi. DNA and RNA levels were quantified by qPCR and normalized to IN+ HIV-1-infected CEM-SS Cas9 cells at 1 dpi, which was set to 1. Mean ± s.d. of 3 biological replicates. Source data

To identify factors that transcriptionally silence unintegrated HIV-1 DNA, we performed a genome-wide CRISPR/Cas9 knockout screen^14^ in the human CD4+ T cell line CEM-SS. We transduced a CEM-SS subclone that expresses *Streptococcus pyogenes* Cas9 with a lentiviral library expressing ~80,000 single guide RNAs (sgRNAs) targeting 19,114 human genes^15^. Seven days later, we infected these cells with IN− NL-GFPΔEnv^10^, an HIV-1 derivative harbouring a deletion in *env*, the inactivating D64V mutation^16^ in IN, and the green fluorescent protein (GFP) open reading frame in place of *nef*. This virus retains intact copies of the other six HIV-1 genes, including *vpr*. At 48 h post infection (hpi), GFP+ cells were collected by fluorescence activated cell sorting (FACS), the sgRNAs recovered by (polymerase chain reaction) PCR then cloned into the same lentiviral vector. After three rounds of selection for GFP+ cells, the sgRNAs were sequenced and analysed for enrichment compared to the starting sgRNA library. As shown in the volcano plot in Fig. 1a, we identified 9 genes that were enriched >16-fold and had a *P* value <0.0005. These included 3 cell surface receptors (LY9, OR52N2 and SSTR2) and 1 motor protein (MYO1B) which were not further analysed. This analysis also recovered 4 of the 8 known components of the SMC5/6 complex, namely SMC5, SMC6, SLF1 and NSMCE3, as well as the DNA repair protein SWI5 (Fig. 1a).

### Validation of the role of SMC5/6 components in silencing HIV-1 DNA

**Extended Data Fig. 1:**
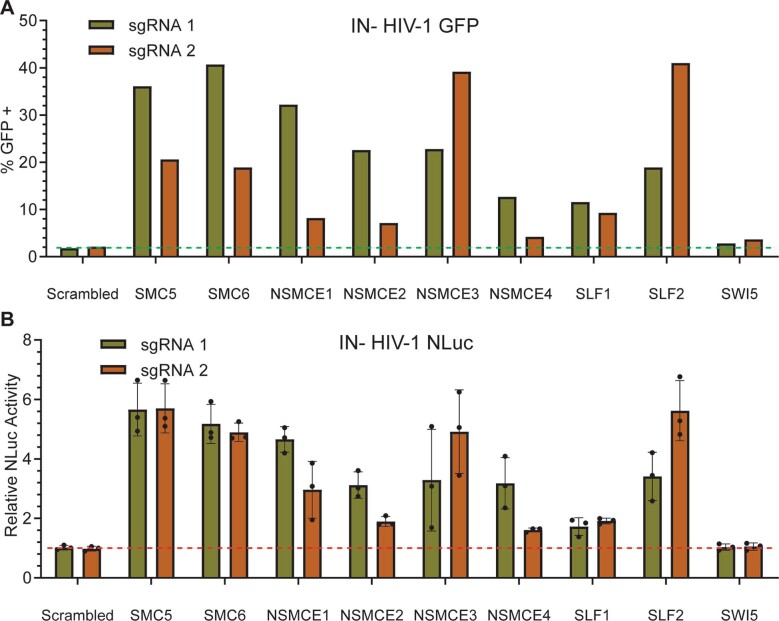
CEM-SS Cas9 cells infected with IN- HIV-1 show increased viral gene expression when any component of the SMC5/6 complex is knocked down. (**a-b**) Polyclonal populations of CEM-SS Cas9 cells transduced with 2 different sgRNAs specific for the indicated genes and then infected with (A) IN- NL-GFPΔEnv, and (B) IN- NL-NLucΔEnv reporter virus. %GFP + cells in A were determined by flow cytometry at 2dpi. NLuc expression in B was normalized to CEM-SS Cas9 cells transduced with a scrambled sgRNA, which was set to 1.0. Data shown in panel B represent the average of three biological replicates with SD indicated. Source data

To confirm the importance of these five proteins, as well as the other four known SMC5/6 complex components (NSMCE1, 2 and 4, and SLF2), we inhibited their expression in CEM-SS Cas9 cells using two independent sgRNAs (Extended Data Fig. 1). Knock down of any of the eight SMC5/6 components substantially enhanced GFP expression from the IN− NL-GFPΔEnv vector. As knock down of SWI5 had no effect, this gene was considered to be a false positive.

**Extended Data Fig. 2:**
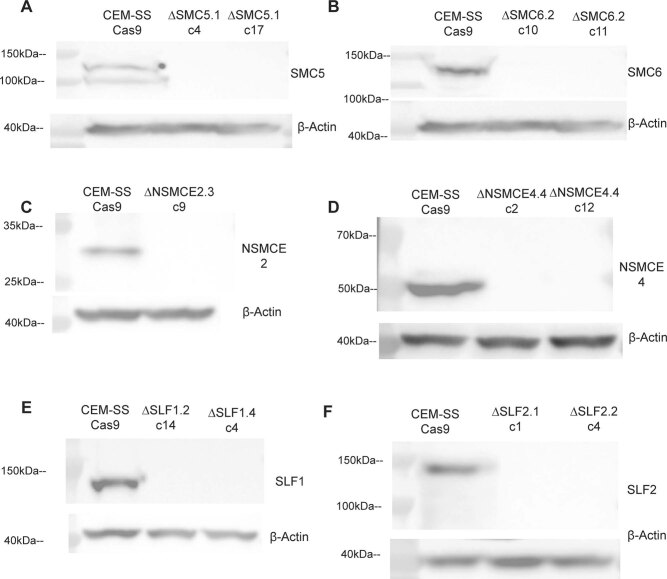
Western Blot analysis confirms the absence of the indicated SMC5/6 complex components in clonal CEM-SS knock out cell lines. Each of the indicated clonal knock out cell lines was lysed then subjected to Western blot analysis using commercial antibodies specific for the indicated SMC5/6 complex component. Actin was used as a loading control. Representative blots shown here are indicative of at least 3 biological replicates. Source data

**Extended Data Fig. 3:**
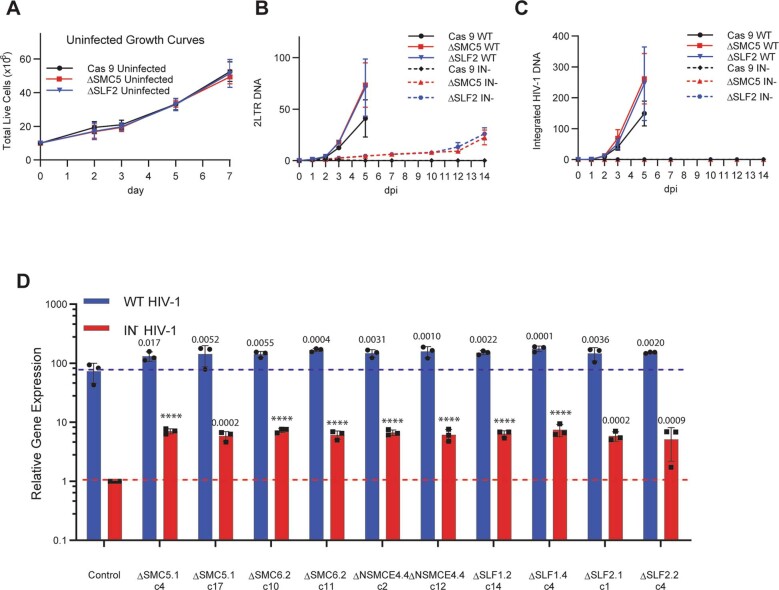
Individual clonal knockout cell lines for the indicated SMC5/6 complex components exhibit similar increases in gene expression from IN- HIV-1 and similar growth rates. (**a**) 7-day growth curves for uninfected CEM-SS Cas9 cells, or the ∆SMC5 and ∆SLF2 CEM-SS clones, counting total live cells in culture. (**b, c**) The indicated cell lines were infected with IN + / IN- HIV-1 and the levels of (B) unintegrated 2LTR DNA, and (**c**) integrated HIV-1 DNA were quantified by qPCR and Alu-qPCR respectively. DNA levels were normalized to IN + HIV-1-infected CEM-SS Cas9 cells at 1dpi, which was set to 1. Data shown represent the average of three biological replicates with SD indicated. (**d**)The indicated clonal knockout CEM-SS cell lines were infected with IN + or IN- NL-NLucΔEnv and viral NLuc expression then assayed at 2 dpi. NLuc levels are given relative to WT CEM-SS Cas9 cells infected with IN- NL-NLucΔEnv (Control), which was set at 1.0. Average of three biological replicates with SD indicated. Data was analyzed using one-way ANOVA, Fisher’s LSD test (p-values indicated on graph, ****p < 0.0001). Source data

We next generated clonal knockout cell lines in CEM-SS cells, for SMC5, SMC6, NSMCE2, NSMCE4, SLF1 and SLF2. In each case, except NSMCE2, we generated two independent knockout cell lines to avoid possible clonal variation. These gene knockouts were verified by identifying inactivating frameshift mutations by DNA sequencing (Supplementary Table 1) and by western blot (Extended Data Fig. 2). The mutant cells showed the same growth kinetics as wildtype (WT) CEM-SS cells (Extended Data Fig. 3). Infection of these cell lines using the IN− NL-NLucΔEnv reporter, in which GFP was replaced with nano luciferase (NLuc)^10^, revealed partial rescue of NLuc expression from unintegrated HIV-1 DNA (Extended Data Fig. 3d). Moreover, infecting these knockout clones with the IN− NL-GFPΔEnv virus at a multiplicity of infection (MOI) of ~0.3 revealed a similar level of GFP+ cells (from 29% to 43% positive, Fig. 1b) to that seen in WT CEM-SS cells infected with an IN+ form of NL-GFPΔEnv (32% positive, Fig. 1c), although the IN+ virus induced a higher mean fluorescence intensity. Infection of ΔSMC5 CEM-SS cells with the IN+ form of NL-GFPΔEnv yielded many more GFP+ cells compared with WT CEM-SS cells (Fig. 1c vs 1d). As HIV-1 proviral integration is inefficient^17^, we hypothesize that this increase results from transcription of unintegrated IN+ HIV-1 DNA. Infection of WT CEM-SS cells with the IN− NL-GFPΔEnv virus did not generate any GFP+ cells (Fig. 1b).

We wondered whether cells lacking SMC5/6 complex function would support the replication of IN− HIV-1. As shown in Fig. 1e–h, IN− HIV-1 bearing the inactivating D64V integrase mutation indeed established spreading infections in the ΔSMC5 and ΔSLF2 subclones, achieving substantial levels of viral DNA, RNA and protein expression by 14 d post infection (dpi) when grown in the presence of raltegravir to prevent any revertant mutations. No replication of IN− HIV-1 was observed in WT CEM-SS cells (Fig. 1f–h). IN− HIV-1 replication in cells lacking functional SMC5/6 complexes caused viral cytopathicity that killed the entire culture by 16 dpi (Fig. 1e). Importantly, at 14 dpi, the inactivating D64V IN mutation was fully retained. Moreover, while 2 long terminal repeat (2LTR) circles characteristic of unintegrated HIV-1 DNA were detected in cultures infected by both IN+ and IN− virus, integrated HIV-1 detected by quantifying integrations into Alu-repeat regions in the genome by Alu-based quantitative PCR (Alu-qPCR)^18^ was only present in the former (Extended Data Fig. 3b,c). Thus, while the replication of IN− HIV-1 in cells lacking the SMC5/6 complex is certainly slower than IN+ HIV-1 (Fig. 1f–h), it is remarkable that IN− HIV-1 can replicate at all.

### The SMC5/6 complex silences unintegrated HIV-1 DNA

**Extended Data Fig. 4:**
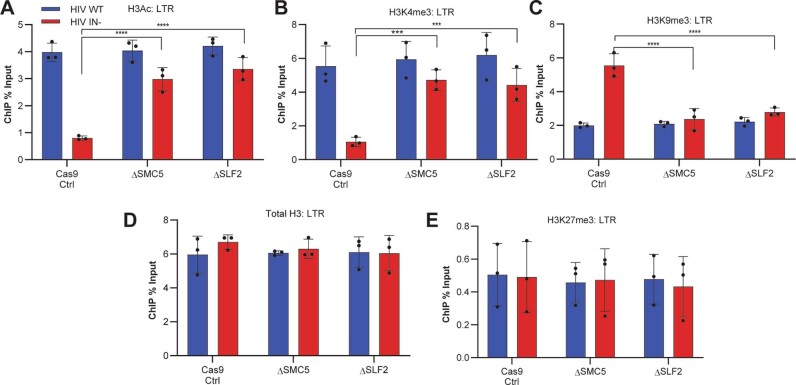
Histone H3 and NSMCE2 binding to HIV-1 DNA. The indicated cell lines were infected with IN + and IN- NL-GFP and subjected to ChIP–qPCR at 2 dpi to quantify the levels of the activating histone modifications H3Ac and H3K4me3 and repressive modifications H3K9me3 and H3K27me3 bound to viral LTR DNA. (**a**) H3Ac and (**b**) H3K4me3 (**c**) H3K9me3, (**d**) total histone H3, and (**e**) H3K27me3. Average of three biological replicates with SD indicated. Data was analyzed with 2-way ANOVA, Sidak’s test (****p < 0.0001; IN- H3K4me3 ΔSMC5: ***p = 0.0009; IN- H3K4me3 ΔSLF2: ***p = 0.0007). Source data

Epigenetic repression of unintegrated HIV-1 DNA is correlated with the addition of the repressive histone modification H3K9me3 and loss of the activating modifications H3Ac and H3K4me3^4,10^. We therefore tested whether loss of the SMC5/6 complex would prevent epigenetic silencing. Analysis of the addition of the activating H3Ac and H3K4me3 modifications to unintegrated IN− HIV-1 DNA revealed a level similar to that seen with IN+ HIV-1 in the ΔSMC5 and ΔSLF2 subclones (Extended Data Fig. 4a,b). Similarly, cells lacking the SMC5/6 complex showed lost inhibitory H3K9me3 modifications on unintegrated HIV-1 DNA (Extended Data Fig. 4c). In contrast, neither the level of total histone H3 binding to viral DNA (Extended Data Fig. 4d) nor the level of H3K27me3 (Extended Data Fig. 4e) were appreciably affected.

**Extended Data Fig. 5:**
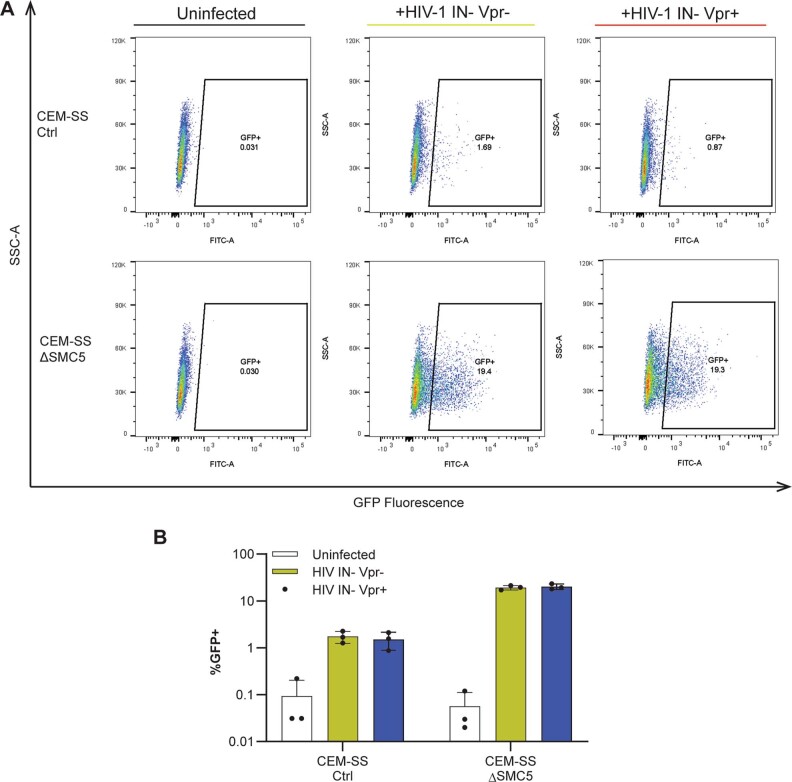
HIV-1 Vpr does not increase viral gene expression from unintegrated HIV-1. CEM-SS Control and ΔSMC5 cells were infected with equal amounts of Vpr+ and Vpr-versions of the IN- NL-GFPΔEnv indicator virus, as measured by ELISA, and GFP expression analyzed by flow cytometry at 3dpi. (**a**) Fluorescence profiles from a representative experiment. (**b**) Quantification of the means from 3 independent biological replicates with SD indicated. Source data

Although the HIV-1 Vpr protein has been reported to enhance gene expression from unintegrated HIV-1 DNA^9,19^ and degrade the SMC5/6 component SLF2^9^, we have previously reported that Vpr overexpression does not enhance gene expression from IN− HIV-1^10^, and WT Vpr+ viruses are strongly inhibited by integrase inhibitors^3^. To test the effect of Vpr on unintegrated viral gene expression, we infected WT or ΔSMC5 CEM-SS cells with the IN− NL-GFPΔEnv virus ±Vpr and saw no discernable difference (Extended Data Fig. 5).

### SUMOylation triggers silencing of unintegrated HIV-1 DNA

**Fig. 2:**
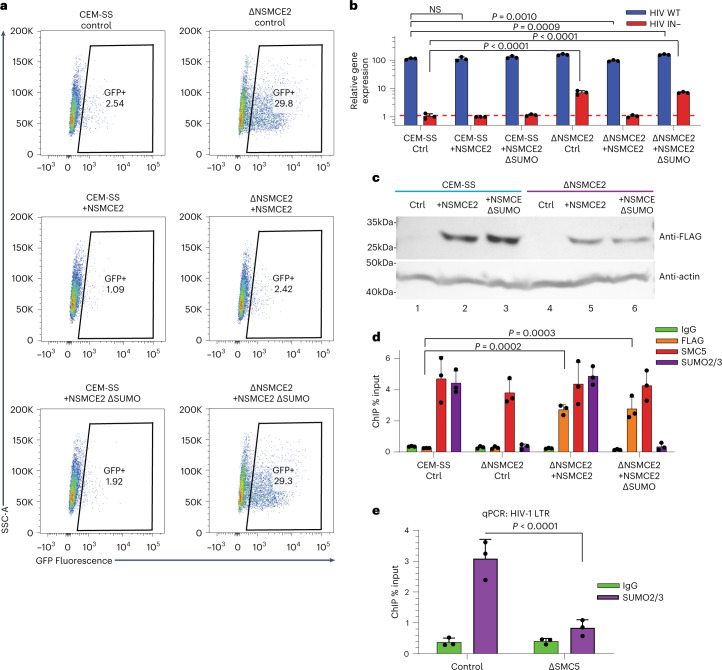
SUMOylation of chromatinized unintegrated HIV-1 DNA by NSMCE2. **a**, Flow cytometry at 2 dpi of WT or ∆NSMCE CEM-SS T cells transduced with a lentiviral vector expressing nothing, FLAG-NSMCE2 or the FLAG-NSMCE2∆SUMO mutant and then infected with IN− NL-GFPΔEnv at an MOI of ~0.3. Shown is a representative experiment from 3 biological replicates. **b**, Quantification of virally encoded NLuc expression in the indicated cells infected with IN+ or IN− NL-NLucΔEnv. NLuc expression was normalized to the IN− HIV-1 infection of WT CEM-SS cells, which was set to 1 (**P = 0.0010, ***P = 0.0009, ****P < 0.0001, 1-way analysis of variance (ANOVA), Dunnett’s test). **c**, Representative western blot of FLAG-tagged WT FLAG-NSMCE and the NSMCE2∆SUMO expression after transduction of the indicated cell types. **d**, ChIP–qPCR quantification of the level of bound, ectopically expressed FLAG-tagged NSMCE2 or endogenous SMC5 to unintegrated IN− HIV-1 DNA in WT or ∆NSMCE CEM-SS T cells. SUMO2/3 deposition was also determined by ChIP–qPCR (ΔNSMCE2 + NSMCE2 ***P = 0.0002, ΔNSMCE2 + NSMCE2ΔSUMO ***P = 0.0003; 2-way ANOVA, Dunnett’s test)**. e**, Quantification of the level of SUMO2/3 deposition on the HIV-1 LTR at 2 dpi in WT and ∆SMC5 CEM-SS cells infected with IN− NL-GFPΔEnv. IgG served as the negative control (****P < 0.0001, 2-way ANOVA, Sidak’s test). Data in **b**, **d** and **e** are mean ± s.d. of 3 biological replicates. Source data

The SMC5/6 complex SUMOylates several protein substrates, including itself^13^, via the SUMO E3 ligase NSMCE2^20,21^. SUMOylation activity, which is stimulated by DNA binding^22^, is essential for the SMC5/6 complex’s role in DNA repair and recombination^13^. Several chromatin components including histone H4 can be SUMOylated and histone SUMOylation is associated with transcriptional repression^23,24^. To determine whether chromatin SUMOylation by SMC5/6 contributes to the epigenetic silencing of unintegrated HIV-1 DNA, we knocked out NSMCE2 in CEM-SS cells as confirmed by DNA sequencing (Supplementary Table 1) and western blotting (Extended Data Fig. 2). Loss of NSMCE2 rescued GFP expression from the IN− NL-GFPΔEnv virus. This rescue was blocked upon ectopic expression of WT NSMCE2 but not the NSMCE2ΔSUMO mutant, which lacks SUMOylation activity due to C185S/H187Q mutations introduced into the essential RING finger domain^25^ (Fig. 2a). A similar result was observed with the IN− NL-NLucΔEnv reporter virus in that loss of NSMCE2 expression in the ΔNSMCE2 subclone rescued NLuc expression from unintegrated HIV-1 DNA and this rescue was blocked by expression of WT but not mutant NSMCE2 (Fig. 2b). This was not due to instability of the NSMCE2ΔSUMO mutant (Fig. 2c). Moreover, both the WT and ΔSUMO forms of NSMCE2 bound unintegrated HIV-1 DNA and loss of NSMCE2 expression did not inhibit the recruitment of the SMC5 component of the SMC5/6 complex to viral DNA (Fig. 2d). Chromatin immunoprecipitation (ChIP)–qPCR using an antibody specific for SUMO2/3 in WT CEM-SS, ΔNSMCE2 and ΔSMC5 cells infected with IN− NL-GFP readily detected the SUMO modification on unintegrated chromatinized HIV-1 DNA in WT cells but not in cells lacking either NSMCE2 or SMC5, which showed background levels of antibody binding (Fig. 2d,e).

**Fig. 3:**
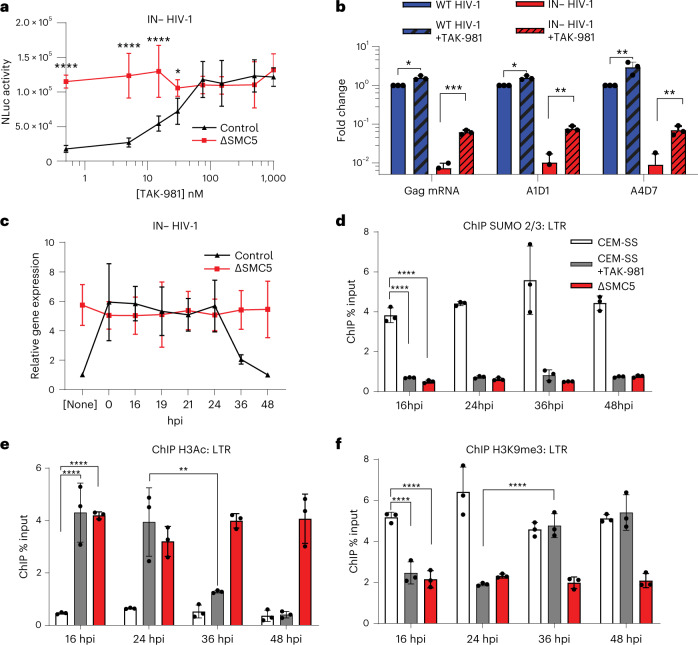
SUMOylation triggers the silencing of unintegrated HIV-1 DNA. **a**, Viral NLuc expression at 2 dpi in WT and ∆SMC5 CEM-SS cells infected with IN− NL-NLucΔEnv and treated with the indicated concentrations of TAK-981 from day 0 (****P < 0.0001, *P = 0.015; 2-way ANOVA, Sidak’s test). **b**, Viral RNA levels for unspliced (Gag mRNA), singly spliced (A1D1) and multiply spliced (A4D7) RNA in CEM-SS cells infected with IN+ or IN− HIV-1 ±150 nM of TAK-981, measured at 2 dpi. RNA levels were normalized to WT HIV-1 infections without TAK-981, which was set to 1, and statistics were calculated using a 2-tailed unpaired Welch *t*-test (Gag: *P = 0.037, ***P = 0.004; A1D1: *P = 0.033, **P = 0.0012; A4D7: **P = 0.0059, **P = 0.0078). **c**, Viral NLuc expression at 3 dpi in WT and ∆SMC5 CEM-SS cells infected with IN− NL-NLucΔEnv, with 150 nM TAK-981 added at the indicated hpi. NLuc expression is given relative to WT CEM-SS cells not treated with TAK-981, which was set to 1. **d**–**f**, ChIP–qPCR to quantify the amounts of SUMO2/3 (**d**), H3Ac (**e**) and H3K9me3 (**f**) deposited on the HIV-1 LTR at 3 dpi. TAK-981 (150 nM) was added at the indicated hpi. Data from **d**–**f** were analysed using 2-way ANOVA, Tukey’s test (****P < 0.0001, **P = 0.0012). Data in **a**–**f** are the mean ± s.d. of 3 biological replicates. Source data

If chromatin SUMOylation is crucial for the epigenetic silencing of unintegrated HIV-1 DNA, then drugs that inhibit SUMOylation should rescue gene expression by IN− HIV-1. The anti-cancer drug TAK-981 is a specific inhibitor of the SUMO-activating enzyme, which catalyses the first step in protein SUMOylation^26^. We performed a TAK-981 dose-response experiment in WT CEM-SS cells as well as in the ΔSMC5 subclone after infection with the IN− NL-NLucΔEnv reporter. Levels of TAK-981, from 5 nM to 1,000 nM, were added at 0 dpi, the cells lysed and NLuc levels quantified at 2 dpi. TAK-981 indeed boosted NLuc expression from unintegrated HIV-1 DNA, reaching a plateau at ~75 nM where the level of NLuc expression from the IN− NL-NLucΔEnv vector in WT cells equalled the level induced in ΔSMC5 cells (Fig. 3a). In contrast, TAK-981 had no effect on gene expression from the IN− NL-NLucΔEnv reporter in ΔSMC5 cells at all doses tested, arguing that the positive effect of TAK-981 on gene expression from unintegrated HIV-1 DNA in WT CEM-SS cells was entirely due to inhibition of SMC5/6 function.

To address the effect of TAK-981 on HIV-1 mRNA expression, we used qPCR to quantify the level of unspliced, singly spliced and fully spliced HIV-1 transcripts in WT CEM-SS T cells infected with WT or IN− HIV-1 in the presence or absence of 150 nM TAK-981 (Fig. 3b). TAK-981 modestly but significantly increased the expression of viral RNA species by IN+ HIV-1 and strongly boosted expression of all three classes of viral RNA from IN− HIV-1.

To determine whether the time of addition of TAK-981 would differentially affect viral gene expression from unintegrated HIV-1 DNA, we infected WT or ΔSMC5 CEM-SS cells with IN− NL-NLuc ΔEnv and then added TAK-981 at different time points from 0 to 48 hpi (Fig. 3c). At 72 hpi, the cells were lysed and NLuc activity determined. Addition of TAK-981 at 0 hpi rescued NLuc expression from the IN− NL-NLucΔEnv reporter virus to the level seen in the ΔSMC5 subclone and this remained true until 24 hpi. However, rescue of NLuc expression by TAK-981 was weaker by 36 hpi and undetectable at 48 hpi. To address whether addition of TAK-981 indeed inhibited SUMOylation of unintegrated chromatinized HIV-1 DNA, we performed ChIP–PCR to determine the level of SUMO present on the viral LTR in WT CEM-SS cells, in the presence and absence of 150 nM TAK-981, and in the ΔSMC5 clone, at 3 dpi with IN− NL-NLucΔEnv. TAK-981 addition resulted in the loss of SUMO from unintegrated HIV-1 DNA regardless of whether the drug was added early or late after infection (Fig. 3d). Thus, even though TAK-981 is ineffective at rescuing gene expression from unintegrated HIV-1 DNA if added at 36 hpi or later (Fig. 3c), it remains effective at preventing the maintenance of the dynamic SUMO modification on viral DNA (Fig. 3d). These data suggest that SUMOylation by SMC5/6 triggers the epigenetic silencing of unintegrated HIV-1 DNA but is not required to maintain the silenced state. This hypothesis was further supported by ChIP–qPCR analysis of the epigenetic modifications present on unintegrated viral DNA in the presence and absence of TAK-981 (Fig. 3e,f). Addition of 150 nM TAK-981 at 16 hpi or 24 hpi rescued addition of the activating H3Ac epigenetic modification to unintegrated chromatinized HIV-1 DNA and blocked addition of the repressive H3K9me3 marker. In contrast, by 36 hpi, TAK-981 was ineffective at reversing the epigenetic silencing of unintegrated HIV-1 DNA. Thus, TAK-981 can prevent the epigenetic repression of IN− HIV-1 if applied early after infection but is unable to rescue pre-existing, epigenetically silenced IN− HIV-1 proviruses and therefore does not function as a latency reversing agent (LRA).

### The epigenetic silencing of integrated HIV-1 proviruses

**Fig. 4:**
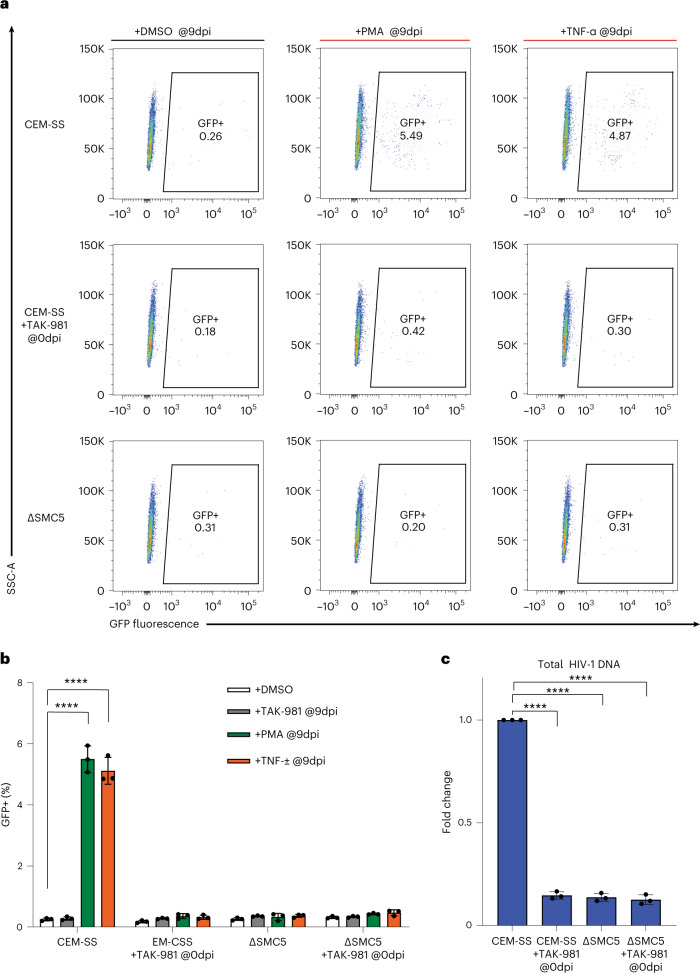
Loss of SUMOylation reduces HIV-1 latency in CD4+ T cells. WT or ΔSMC5 CEM-SS cells were infected with IN+ NL-GFPΔEnv ±TAK-981. At 3 dpi, GFP− cells were isolated by FACS (see Extended Data Fig. 6a for the relevant FACS profiles) and the cells then cultured for an additional 6 d to allow all unintegrated HIV-1 DNA to be lost (Extended Data Fig. 6b) before being treated either with diluent dimethyl sulfoxide (DMSO), TAK-981 or the LRAs PMA or TNF-α. One day later at 10 dpi, green fluorescent protein (GFP) expression was analysed by FACS. **a**, Representative FACS profiles. **b**, A summary of data in **a** showing the mean ± s.d. of 3 biological replicates (****P < 0.0001, 2-way ANOVA, Tukey’s test). **c**, Total HIV-1 DNA was quantified in the indicated cells at 9 dpi before drug addition. Data are the mean ± s.d. of 3 biological replicates (****P < 0.0001, 1-way ANOVA, Dunnett’s test). Source data

**Extended Data Fig. 6:**
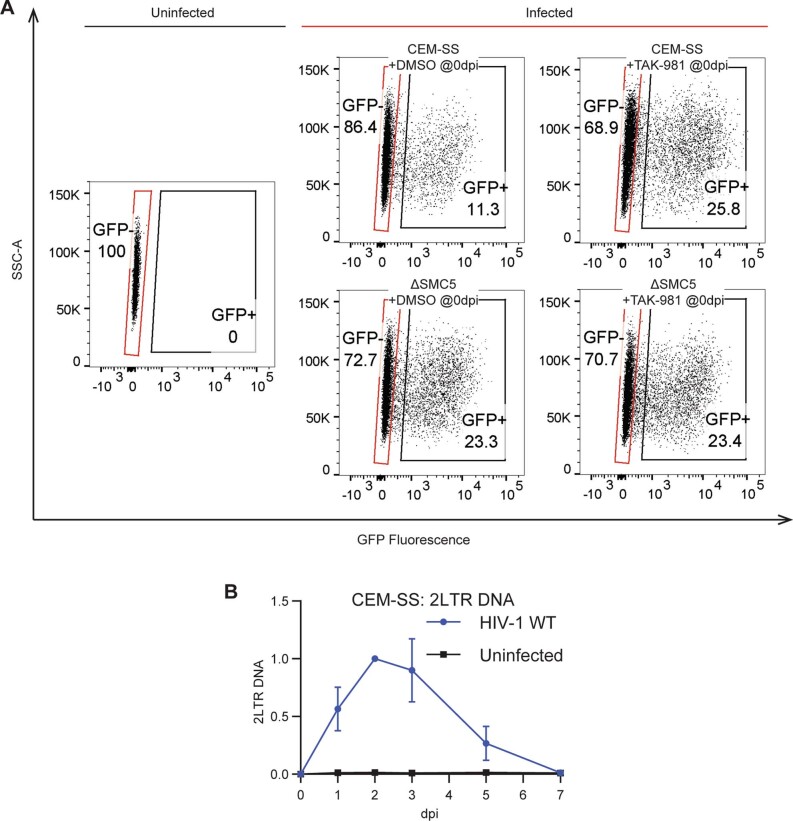
Loss of the SMC5/6 complex boosts HIV-1 gene expression. (**a**) WT or ΔSMC5 CEM-SS cells were infected with VSV-G pseudotyped IN + NL-GFPΔEnv in the presence or absence of TAK-981 at an MOI of ~0.1. Cells were sorted by FACS at 3dpi and GFP- cells (gated in red) harvested for the experiments reported in Fig. 4a. (**b**) The level of unintegrated HIV-1 2LTR DNA was quantified by qPCR in CEM-SS cells infected with WT HIV-1 until 7dpi, when it declined to background levels. Average of 3 biological replicates with SD indicated. Data were normalized to the signal at 2dpi, which was set to 1.0. Source data

It was recently proposed that infection with IN+ HIV-1 could generate proviruses that had been epigenetically silenced before integration and then remained silenced post-integration, thus generating latently infected T cells^27^. If correct, then inhibiting the silencing of unintegrated HIV-1 DNA by SMC5/6 should also inhibit the formation of latently infected T cells. To test this hypothesis, we asked whether loss of SMC5/6 expression or treatment with TAK-981 at 0 dpi would inhibit the establishment of latent HIV-1 infections in CEM-SS T cells. The assay used^28^ involves the infection of WT or ΔSMC5 CEM-SS cells with the IN+ NL-GFPΔEnv indicator virus in the presence or absence of TAK-981 at an MOI of ~0.1. At 3 dpi, the cells were subjected to FACS and GFP-negative cells representing either uninfected or latently HIV-1 infected cells were isolated. The FACS profiles again showed a higher level of GFP+ cells in the WT CEM-SS treated with TAK-981 at 0 dpi and in the ΔSMC5 CEM-SS regardless of TAK-981 treatment (Extended Data Fig. 6a), presumably again resulting from transcription of unintegrated IN+ HIV-1 proviruses. The isolated GFP-negative cells were cultured for an additional 6 d and then treated with diluent dimethyl sulfoxide (DMSO) or with TAK-981, phorbol myristate acetate (PMA) or tumour necrosis factor alpha (TNF-α). Both PMA and TNF-α are potent activators of NF-kB activity and effective LRAs^28,29^. One day later at 10 dpi, the cells were again analysed by FACS (a representative experiment is shown in Fig. 4a and a compilation of three independent biological replicates is shown in Fig. 4b). Importantly, all unintegrated HIV-1 DNA was lost from infected CEM-SS cells by 7 dpi (Extended Data Fig. 6b), so any GFP expression seen at 10 dpi must originate from integrated proviruses.

In WT CEM-SS cells, PMA and TNF-α both induced GFP expression in ~5% of the sorted GFP-negative cells, while the level of GFP+ cells in the uninduced culture was ~0.3% (Fig. 4a,b), a difference that is highly significant (*P* < 0.001). In contrast, PMA or TNF-α treatment at 9 dpi failed to induce GFP expression above background in WT CEM-SS cells treated at 0 dpi with TAK-981, or in the ΔSMC5 CEM-SS clone either in the absence or presence of TAK-981 treatment at 0 dpi (Fig. 4a,b), even though the initial infection of these cells, as measured by GFP expression, was actually higher than seen in WT CEM-SS cells (Extended Data Fig. 6a).

If loss of SMC5/6 complex function indeed inhibits the establishment of latent HIV-1 infections, then the FACS-sorted WT GFP− cells (Extended Data Fig. 6) should contain more latent HIV-1 proviral DNA than the GFP− cells that lacked SMC5/6 function at the time of infection. In fact, qPCR analysis performed at 9 dpi before drug addition demonstrated that the isolated WT GFP− cells indeed contained significantly (*P* < 0.001) more HIV-1 DNA that did GFP− ΔSMC5 cells or WT cells treated with TAK-981 at the time of infection (Fig. 4c).

### Delayed integration increases the incidence of HIV-1 latency

**Extended Data Fig. 7:**
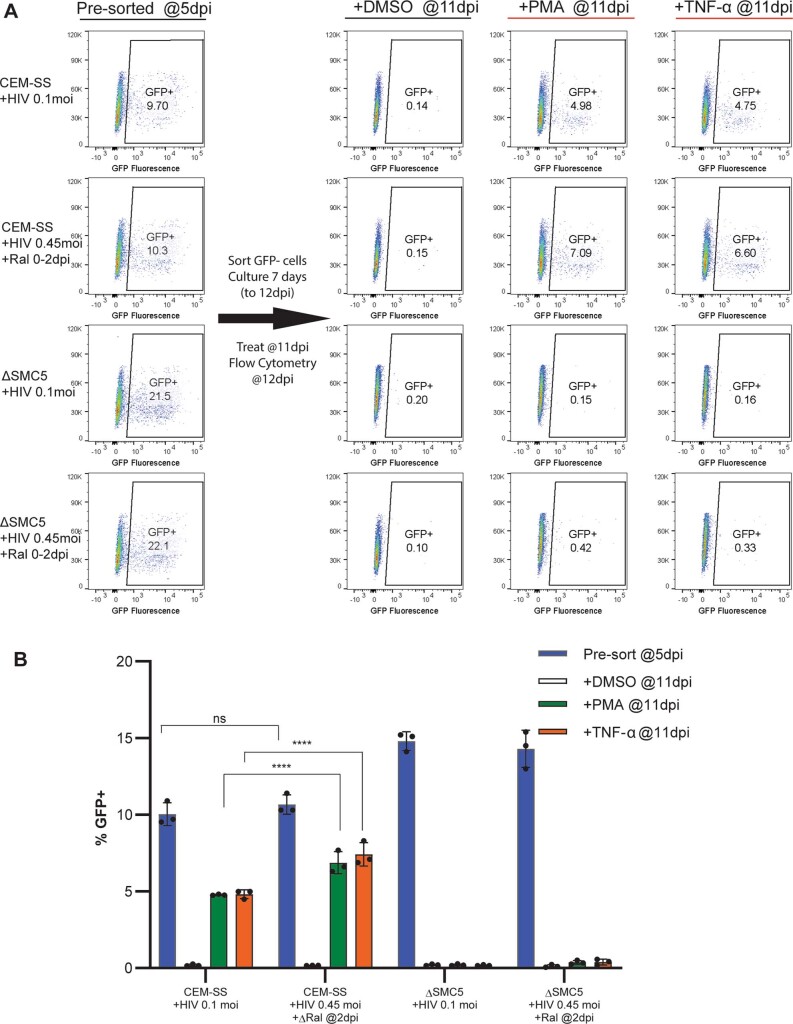
Extending the pre-integration stage of infection with raltegravir increases the percentage of latently infected cells. WT or ΔSMC5 CEM-SS cells were infected with IN + NL-GFPΔEnv in the presence and absence of the integrase inhibitor raltegravir. These cells were infected at a multiplicity of infection (MOI) such that the GFP + cells in both the +ral and -ral were roughly identical when sorted at 5dpi, as indicated in panel A. At 2dpi, the cells were washed to remove raltegravir and replated to allow integration to occur. GFP- cells were isolated by FACS at 5dpi and cultured for another 6 days (11dpi) where they were treated with diluent (DMSO), TAK-981, PMA, or TNF-α. The cells were then analyzed by flow cytometry at 12dpi for GFP expression. (**a**) Representative GFP expression profiles from a single experiment. (**b**) Compilation of 3 independent biological replicates, showing the means and with SD indicated. Data analyzed by 2-way ANOVA, Tukey’s test (****p < 0.0001). Source data

If epigenetic silencing of unintegrated HIV-1 DNA can lead to the establishment of latent infections, then delaying integration might increase the number of latent infections. To test this idea, we infected WT CEM-SS cells with the IN+ form of the NL-GFPΔEnv indicator virus in the presence or absence of the integrase inhibitor raltegravir. The drug was washed out at 2 dpi and GFP− cells isolated by FACS at 5 dpi. The cells were then treated with diluent, PMA or TNF-α at 11 dpi and analysed by FACS at 12 dpi. Treatment with raltegravir from 0 to 2 dpi indeed increased the percentage of cells that expressed GFP after treatment with either PMA or TNF-α, even though the number of GFP+, that is, productive infections detected at 5 dpi was similar (Extended Data Fig. 7). Thus, prolonging the time between proviral DNA synthesis and integration significantly (*P* < 0.001) increases the number of latent integrated proviruses that can then be activated by an LRA. These data demonstrate that HIV-1 latency can indeed be established before proviral integration.

### SUMOylation facilitates HIV-1 latency in primary T cells

**Fig. 5:**
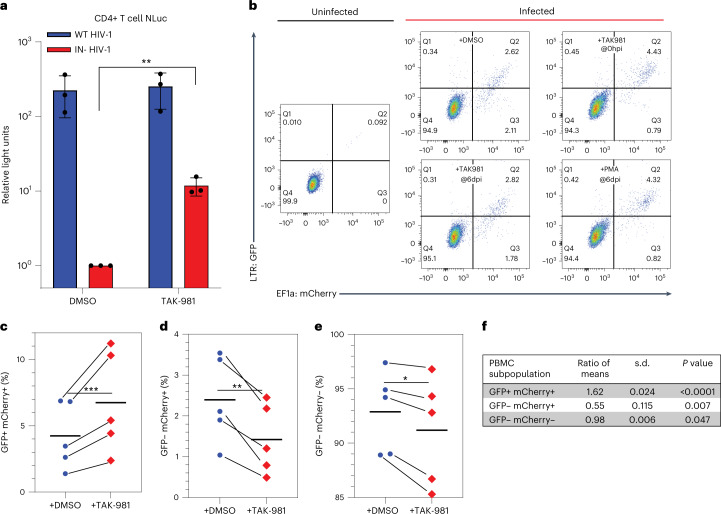
Epigenetic silencing of unintegrated HIV-1 DNA leads to latent infections. **a**, CD4+ T cells isolated from blood were infected with IN+ or IN− NL-NLucΔEnv in the presence of 150 nM TAK-981 in DMSO or with only DMSO, at 0 hpi. Cells were collected at 48 hpi, lysed and NLuc levels determined. Mean ± s.d. of 3 biological replicates (**P = 0.0045, 2-tailed unpaired *t*-test). **b**, CD4+ T cells were infected with a replication-defective, GFP/mCherry dual-colour reporter virus at a low MOI of ~0.05 in the presence of DMSO or 150 nM TAK-981 added at infection, or with 150 nM TAK-981 or 80 nM PMA added at 6 dpi. The number of GFP and mCherry expressing cells was then determined at 7 dpi by FACS. **c**–**e**, Data from 5 independent primary CD4+ T cell infections carried out as in **b** showing the percentage of GFP+ mCherry+ (**c**), GFP− mCherry+ (**d**) or GFP− mCherry− (**e**) cells at 7 dpi in the presence and absence of TAK-981 added at 0 dpi. Individual experiments using the same T cell samples ±TAK-981 are linked by lines. **f**, Statistical analysis of ratios from the data shown in **c**–**e** using a 2-tailed ratio paired *t*-test. Source data

**Extended Data Fig. 8:**
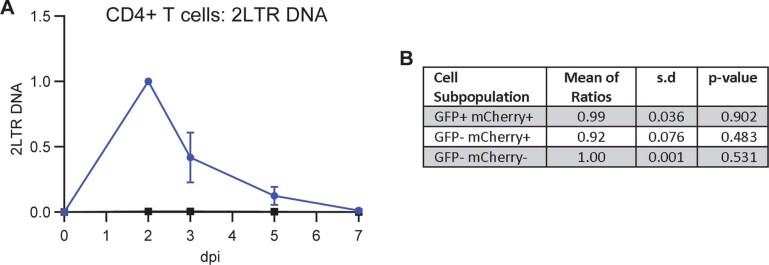
The SMC5/6 promotes HIV-1 latency. (**a**) In WT HIV-1 infected primary CD4 + T cells, unintegrated HIV-1 DNA peaks at 2dpi and declines to background levels by 7dpi, as measured by qPCR analysis of 2LTR circular viral DNA. These data represent the average of five biological replicates using CD4 + T cells obtained from five separate blood donors, with SD indicated. Data were normalized to the signal detected at 2dpi, which was set at 1.0. (**b**) Statistical analysis of the indicated GFP/mCherry subpopulations in cells infected with the dual color HIV-1 indicator virus and treated with 150 nM TAK-981 at 6dpi vs diluent (DMSO) at 0dpi. Infected cells were harvested at 7dpi and analyzed by FACS. Statistics calculated with a 2-tailed ratio paired t-test. Source data

To examine whether SMC5/6 also contributes to the establishment of viral latency in primary cells, we first confirmed that TAK-981 could also rescue gene expression from unintegrated HIV-1 DNA in infected primary CD4+ T cells (Fig. 5a). Next, we asked whether TAK-981 could inhibit the establishment of latent HIV-1 infections in primary CD4+ T cells using an assay for HIV-1 latency that uses a dual-colour reporter virus^30,31^. In these viruses, GFP is expressed under the control of the HIV-1 LTR, which is silenced in latent infections, while mCherry is expressed using the exogenous EF1-α promoter, which is resistant to epigenetic silencing. Infection of T cells with a dual-colour HIV-1 reporter generates cells that are GFP+ and mCherry+, representing productive infections, and cells that are GFP− but mCherry+, representing latent infections. We therefore asked whether addition of TAK-981 at 0 dpi but not at 6 dpi to cells infected with the IN+ dual-colour reporter virus would affect the level of GFP− mCherry+ cells at 7 dpi, which is the earliest time point when all unintegrated HIV-1 DNA would have been lost (Extended Data Fig. 8a).

In fact, we observed a consistent reduction in the number of latently infected, GFP− mCherry+ primary T cells when TAK-981 was added at 0 dpi (from 2.11% to 0.79% in Fig. 5b), but we saw no significant effect on the level of GFP− mCherry+ cells when TAK-981 was added at 6 dpi, as predicted (Fig. 5b, see also Extended Data Fig. 8b). We saw a concomitant increase in the percentage of GFP+ mCherry+ T cells in the culture treated with TAK-981 at 0 dpi (from 2.62% to 4.43%, in Fig. 5b), representing productive infections. Again, addition of TAK-981 at 6 dpi had no effect (Fig. 5b, see also Extended Data Fig. 8b).

As shown in Fig. 5c–e, which present data compiled from five independent biological replicates using primary CD4+ T cells from five different blood donors, we saw a consistent increase in the number of GFP+ mCherry+ T cells (Fig. 5c) and a consistent decrease in the number of GFP− mCherry+ cells (Fig. 5d) in every experiment analysed. Overall, addition of TAK-981 at 0 dpi increased the number of productively infected GFP+ mCherry+ cells by 1.62 ± 0.024-fold (*P* < 0.0001) and decreased the number of latently infected GFP− mCherry+ cells by 0.55 ± 0.115-fold (*P* = 0.007) (Fig. 5f). The increase in GFP+ mCherry+ cells derived not only from the GFP− mCherry+ population but also from the GFP− mCherry− population, which decreased modestly but significantly in the culture treated with TAK-981 at 0 dpi (Fig. 5e,f, overall ratio 0.98 ± 0.006, *P* = 0.047). Therefore, TAK-981 treatment at 0 dpi also inhibits the generation of cells in which both the LTR and EF1-α promoter are epigenetically silenced.

## Discussion

Here we show that the human SMC5/6 complex induces the SUMOylation of chromatinized unintegrated HIV-1 DNA leading to its epigenetic silencing. As a result, loss of SMC5/6 expression, or inhibition of chromatin SUMOylation using the inhibitor TAK-981, rescues gene expression from unintegrated HIV-1 DNA and even allows IN− HIV-1 to establish a spreading infection in cultured T cells (Fig. 1). Surprisingly, we also demonstrate that loss of SMC5/6 expression, or treatment with TAK-981, markedly inhibits the establishment of HIV-1 latency in both the CEM-SS T cell line and in primary CD4+ T cells (Figs. 4 and 5).

Although antiretroviral therapies can reduce the viral load in AIDS patients to below the level of detection, these drugs fail to cure HIV-1. This is due to the continued presence of a small number of latently infected T cells that contain integrated intact HIV-1 proviruses that are transcriptionally inert yet can be activated by external stimuli to rekindle viral replication^32^. While there has been considerable effort expended on trying to develop LRAs that can activate latent HIV-1 and, in the presence of antiretroviral therapies, clear the body of infectious virus, this effort has so far failed to identify LRAs that are both effective and non-toxic.

The latent reservoir has been attributed to activated T cells that are infected by HIV-1 coincident with their reversion to resting memory T cells^32^. However, HIV-1 latency can be established in both CD4 T cell lines and activated T cells in vitro^28,31^, and most of the latent reservoir in patients undergoing antiretroviral therapies is maintained through clonal expansion^33–36^, with many latent cells expressing proliferation markers HLA-DR^37,38^, CD25^39^ and CD69^40^. Thus, repression of HIV-1 gene expression in latency is not simply the result of the quiescent state of resting memory T cells. Latency has also been proposed to result from the integration of HIV-1 proviruses into regions of heterochromatin, resulting in epigenetic silencing^41^. Yet analysis of individual latent HIV-1 integration sites in patients has shown that most of the intact full-length HIV-1 proviruses are integrated into actively transcribed genes^42–46^. Overall, the mechanism(s) underlying the establishment of HIV-1 latency have remained elusive and cellular factors that promote viral latency largely undefined.

Our data suggest that the key mechanism underlying the initiation of HIV-1 latency is the epigenetic silencing by SMC5/6 of unintegrated proviruses that retain their pre-existing inhibitory epigenetic modifications after integration. These data identify the SMC5/6 complex as being directly involved in promoting the establishment of HIV-1 latency and suggest that latency results not from any intrinsic properties of the incoming retrovirus but rather from an unfortunate side effect of a cellular innate immune response that probably evolved to silence invasive foreign DNA.

## Methods

### Cell lines and primary cultures

#### Immortalized cell lines

Human 293T cells (female) were initially purchased from the American Type Culture Collection (ATCC) and were cultured in Dulbecco’s modified Eagle medium (DMEM, Sigma) supplemented with 10% fetal bovine serum (FBS, Hyclone) and an antibiotic-antimycotic (Gibco).

Human CEM-SS cells (female) were obtained from the NIH AIDS Reagent and were cultured in Roswell Park Memorial Institute (RPMI) medium supplemented with 10% FBS and antibiotic-antimycotic.

CEM-SS cells stably expressing Cas9 protein were produced using a modified pLentiCrispr v2-Blast plasmid that was a gift from Mohan Babu (Addgene plasmid 83480). The U6-promoter, sgRNA scaffold and EF1-α promoter were excised from pLentiCrispr v2-Blast by cleavage with KpnI and AgeI and replaced with an SFFV promoter. Lentiviruses were made from this construct by transfecting 5 × 10^6^ 293T cells in a 15 cm dish with 15 µg of the lentiviral vector, as well as 10 µg and 5 µg of the packaging plasmids pCMVR8.74 and pMD2.G, respectively, using polyethylenimine (PEI). The media were changed 24 h post transfection (hpt). Supernatants containing lentiviral particles were collected at 72 hpt, filtered through a 0.44 µm filter and run through a 100,000 MWCO concentrator (Amicon). Following concentration, 5 × 10^6^ CEM-SS cells were incubated with 2 ml of the concentrated supernatant at 37 °C overnight. The media were then replaced with fresh RPMI medium and cells incubated for 48 h. At this point, the media were replaced with fresh RPMI medium supplemented with 20 µg ml^−1^ of blasticidin (Santa Cruz) to allow selection of transduced cells. Cells were then single-cell cloned by aliquoting limited dilution in 96-well plates such that each well has ~10% chance of having a cell in it. These cloned cells were then analysed for Cas9 activity.

#### Primary CD4+ T cells

Human blood from healthy donors was purchased from the Gulf Coast Regional Blood Center. All donors tested negative for HIV-1 and HIV-2. Samples were de-identified before purchasing. Peripheral blood mononuclear cells (PBMCs) were isolated from whole blood by density-gradient centrifugation over Histopaque (Sigma) and CD4+ cells isolated using a CD4+ isolation kit (Invitrogen). Isolated CD4+ cells were activated by incubation with antibodies against CD28/CD49d (BD Biosciences) and 5 µg ml^−1^ phytohaemagglutinin (PHA) in RPMI supplemented with 10% FBS and IL-2 as previously described^47^. Cells were maintained at 10^5^–10^6^ cells per ml in the presence of IL-2, CD28/CD49d antibodies and PHA for 1 week before infection with HIV-1.

### HIV-1 production

An HIV-1 nano luciferase reporter virus (NL-NLuc) was generated from the parental NL4-3 virus by substituting the viral *nef* gene in NL4-3 with the *NLuc* indicator gene^48^. NL-NLucΔEnv was made from NL-NLuc by removal of a 943 bp segment of the *env* gene that makes it replication incompetent. Similarly, the GFP reporter virus (NL-GFPΔEnv) was generated from NL-NLucΔEnv by substituting *GFP* in place of *nef*. The NL-DC reporter virus expresses eGFP under the control of the HIV-1 LTR and mCherry from an internal EF1-α promoter and was generated by cloning an EF1-α:mCherry cassette into the XhoI site located 3′ to GFP in NL-GFPΔEnv. All these reporter viruses have an intact *vpr* gene.

A GFP reporter virus, IN− NL-GFPΔVpr, lacking a functional Vpr protein, was created from the parental IN− NL-GFP virus by inserting a TTAA duplication at 15 bp 3′ of the Vif stop codon. This introduces a stop codon and a frameshift mutation early in the Vpr open reading frame that creates a non-functional truncated Vpr protein^49^. These viruses either have WT integrase (IN+) or contain the D64V (IN−) mutation that blocks IN function.

**Extended Data Fig. 9:**
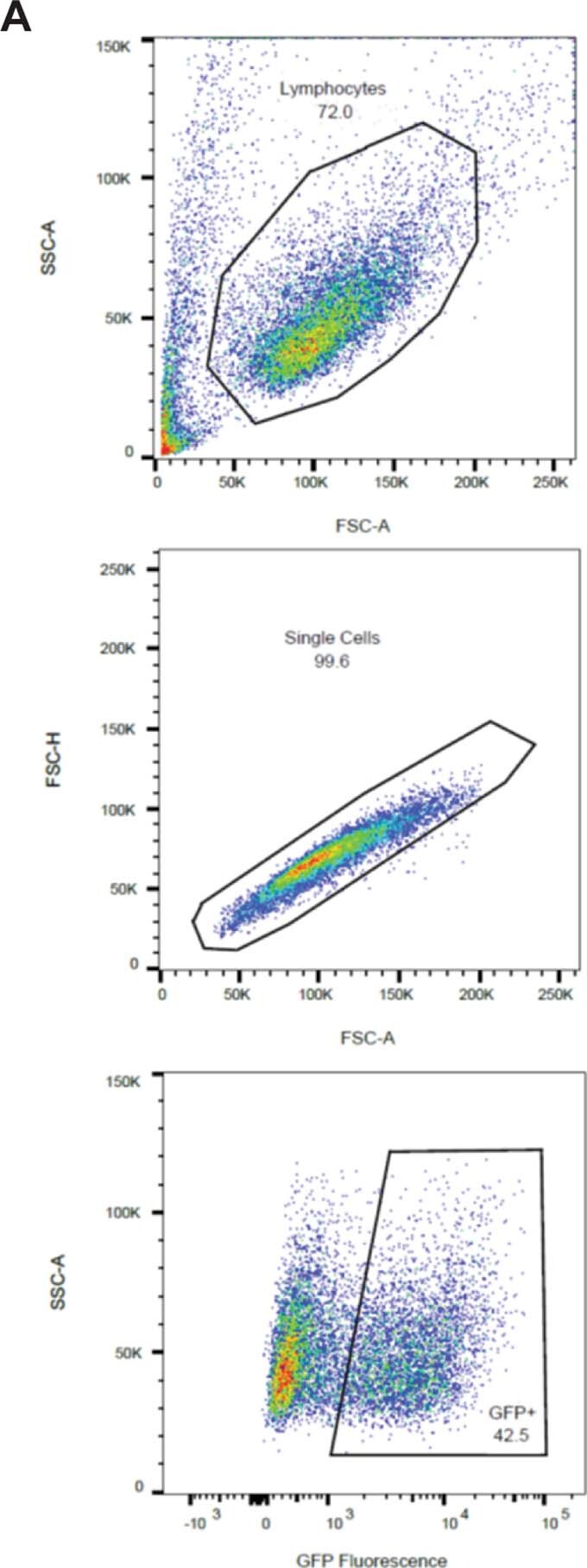
Example flow cytometry gating strategy. Live T cells were gated on forward vs. side scatter plot based on their size and granularity. Single cells were gated on a forward scatter height vs. forward scatter area plot to remove doublets. The gates for GFP + and/or GFP- cells are defined according to the uninfected controls and are evident in the final figures.

Plasmids expressing the replication-competent NL-NLuc provirus were transfected into 293T cells using PEI. Non-spreading NL-NLucΔEnv and NL-GFPΔEnv proviruses were co-transfected into 293T cells with the pMD2.G plasmid encoding the VSV-G protein. After 24 h, the spent media were replaced with fresh media. At 72 hpt, supernatant media were filtered through a 0.44 μm filter. WT or IN− HIV-1-containing supernatant media were normalized by p24 levels, measured by ELISA, before being used to infect target cells. The MOI:p24-normalized volume ratio was assessed by infecting 10^6^ CEM-SS cells with IN+ NL-GFPΔEnv, or a CEM-SS inducible Tax cell line (expressing an HTLV-1 Tax protein that is known to activate gene expression from unintegrated HIV-1^10^) with IN− NL-GFPΔEnv. These cells were infected with varying dilutions of the virus, then analysed by flow cytometry at 2 dpi (gating strategy outlined in Extended Data Fig. 9). The number of GFP+ cells at each dilution was then converted to MOI using the formula MOI = −ln (1− proportion of GFP+ cells) which was then correlated to the p24 level in the viral stock. Thus, the amount of IN− virus used (per million cells) for each MOI could be determined.

### CRISPR knockout screen

The Brunello human CRISPR knockout library (Addgene 73178)^15^ was used to transform electrocompetent cells (Endura). Of the library (resuspended in water), 500 ng was used to transform a total of 4 × 25 μl of cells in a 1 mm cuvette (10 μF, 600 Ω, 1.8 kV) to yield >10^8^ colonies when plated to ensure each sgRNA in the library was covered ~1,000× on average. These colonies were collected and the pooled library plasmids extracted using Maxiprep columns (Zymo).

Lentiviral libraries were created by transfecting 293T cells with library DNA and the packaging plasmids pCMVR8.74 and pMD2.G using PEI. The media were changed at 24 hpt, and the supernatant containing the lentivirus library was collected and filtered through a 0.44 µm filter at 72 hpt. The lentivirus was titrated and used to transduce CEM-SS Cas9 cells that were then subjected to puromycin (Gemini) kill curves at 2 dpt to determine the amount of lentivirus that correlated to an MOI of 0.3. This amount was then used to transduce 10^8^ CEM-SS Cas9 cells at 0.3 MOI, and the cells were selected in 1 µg ml^−1^ puromycin at 2 dpt for a week.

The pooled knockout cells were then infected for 2 d with an IN− HIV-1 NL-GFP reporter virus that has an inactivating D64V amino acid substitution in the integrase gene. These infected cells were then run through a BSL-3 contained FACS Aria II (BD Biosciences) to collect the GFP+ cell population from which genomic DNA was extracted. Purified gDNA was incubated with the restriction enzyme DpnI to remove any residual plasmid contamination.

The sgRNA from this DNA was amplified by PCR using flanking primers (FP: 5′-TGGACTATCATATGCTTACCGTAACTTGA -3′ RP: 5′-GGCTCGAGGGGGCCCGGGTGCAAAGATGGATA -3′) and then cloned back into the parental pLentiGuide Puro plasmid via the NdeI and XmaI restriction sites. Subsequent rounds of transformation, lentivirus production, transduction and infection were carried out as described for a total of 3 rounds. In the final round, the DNA was amplified using the indexed Illumina sequencing primers, purified in a PCR purification spin column (Zymo) and sequenced on the NovaSeq 6000.

Sequencing data were analysed using MAGECK-VISPR^50^ by first generating sgRNA read counts via invoking ‘mageck count’, analysing sgRNA enrichment and getting gene ranks using the Robust Rank Aggregation algorithm on normalized read counts. ‘Mageck test’ was run with ‘–remove-zero both–remove-zero-threshold 0’ parameters as previously suggested^51^.

### CRISPR single-gene knockouts

Single-gene knockout cells were generated by transducing CEM-SS Cas9 cells with lentiviruses made from a pLentiGuide-Puro plasmid (Addgene 52963)^52^ expressing the sgRNA of interest. Transduced cells were selected at 2 dpt with 1 µg ml^−1^ puromycin for 1 week. Infection experiments on polyclonal knockout cells were carried out by infecting cells at this stage.

Clonal cells were isolated by aliquoting puromycin-resistant cells at limiting dilution into a 96-well plate, subsequent isolation and expansion. Knockout cells were then identified and validated by clonal sequencing of the genetic lesions and by western blot.

### Western blot analyses

Cells were collected and lysed in Laemmli buffer, sonicated and denatured at 95 °C for 15 min. Lysates were subjected to electrophoresis on 4–20% SDS–polyacrylamide gels (Bio-Rad), transferred onto nitrocellulose membranes and then blocked in 5% milk in PBS + 0.1% Tween. Membranes were incubated in primary and secondary antibodies diluted in 5% milk in PBS + 0.1% Tween for 2 h each and then washed in PBS + 0.1% Tween. The membranes were incubated with a luminol-based enhanced chemiluminescent substrate and signals were visualized using GeneSnap (Syngene). The membranes were immunoblotted with specific antibodies to probe for SMC5 (Research Resource Identifier RRID:AB_2900565), SMC6 (RRID:AB_2747157), NSMCE2 (RRID:AB_10637854), NSMCE4A (RRID:AB_11169701), SLF1 (RRID:AB_10816722), SLF2 (RRID:AB_11129755), FLAG (RRID:AB_259529) or actin (RRID:AB_2687938). Primary antibodies were used at 1:1,000 dilution, except for the actin antibody which was used at 1:5,000. Secondary HRP-conjugated anti-mouse (RRID:AB258431) or anti-rabbit (RRID:AB_258284) antibodies were used at 1:5,000 dilution.

### Flow cytometry

Cells were collected, washed in PBS and fixed in 1% paraformaldehyde in PBS for 10 min before being resuspended in 2% bovine serum albumin (BSA) in PBS and run through a cell strainer. Cells were run through a Fortessa X20 flow cytometer (BD Biosciences) and the data analysed using FlowJo v10.6.2.

### Luciferase assay

Cells were collected, washed three times in PBS, lysed in passive lysis buffer (Promega) and assayed for NLuc activity using the Nano-Glo luciferase assay on a Lumat LB9507 luminometer (Bertold Technologies).

### Quantification of HIV-1 replication and spread

Cells (10^7^) from the parental Cas9 cell line, or the ΔSMC5 and ΔSLF2 cell lines, were infected with either WT or IN− NL-NLuc virus in a total of 20 ml RPMI. Viral stocks were pretreated with 5 U ml^−1^ DNase I to remove residual plasmid DNA, and all IN− infections were carried out in the presence of 20 µM raltegravir to prevent revertant mutations. Cells were counted at days 1, 2, 3, 5, 7, 10, 12, 14 and 16 dpi where possible, and media were periodically refreshed to maintain cell counts below 10^6^ cells per ml. Live cells (10^6^) were collected at each time point (where possible) and equally split to assay NLuc and for DNA and RNA extraction.

For DNA analysis, cells were pelleted and washed three times in ice-cold PBS. DNA was then extracted using DNA Miniprep Plus columns (Zymo) according to the manufacturer’s instructions, then incubated with DpnI (NEB) to remove any residual plasmid contamination.

For RNA analysis, cells were lysed in TRIzol (Thermo Fisher) to collect the RNA, and DNAse I treated to remove residual DNA contamination. The RNA was then converted to complementary DNA using the High Capacity cDNA Reverse Transcription kit (Applied Biosystems).

Quantification of total HIV-1 DNA and RNA was carried out on a QuantStudio 6 Pro real-time qPCR machine (Thermo Fisher) using a custom total HIV-1 *Taq*Man probe that amplifies the U5-gag region on HIV-1.

For Alu-LTR real time nested qPCR, DNA was amplified using a nested PCR approach^18^. Briefly, an initial non-saturating PCR using primers ALU1 (5′-TCCCAGCTACTGGGGAGGCTGAGG-3′), ALU2 (5′-GCCTCCCAAAGTGCTGGGATTACA-3′) and L-HIV (5′- ATGCCACGTAAGCGAAACTTAAGCCTCAATAAAGCTTGC-3′) was performed using DNA isolated from HIV-1 infected cells. After the PCR products were purified using a PCR Kleen kit (Bio-Rad), nested qPCR was performed using primers AA55M (5′- GCTAGAGATTTTCCACACTGACTAA-3′) and L (5′- ATGCCACGTAAGCGAAAC-3′) and the SYBR green master mix (Thermo Fisher).

The amounts of unintegrated 2LTR HIV-1 circular DNA were quantified by qPCR using *Taq*Man primers/probes that amplify across the U5-U3 junction only present in 2LTR circles (FP: 5′- AACTAGGGAACCCACTGCTTAAG -3′, RP: 5′- TCCACAGATCAAGGATATCTTGTC -3′, probe: 5′- FAM- ACACTACTTGAAGCACTCAAGGCAAGCTTT -TAMRA-3′)^53^.

Relative quantification using the ΔΔCT method with β-Actin as an internal control was then carried out using either a genomic β-Actin (DNA) or spliced β-Actin (RNA) probe. Relative quantification using the ΔΔCT method^54^ with β-Actin as an internal control was then carried out.

### ChIP–qPCR

The indicated cells were cultured at 10^6^ cells per ml in RPMI and infected with IN− NL-GFP. Viral stocks were pre-incubated with 5 U ml^−1^ DNase I to remove plasmid contamination before infection.

Cells were collected at the indicated times post infection, rinsed twice with PBS and crosslinked with 1% formaldehyde for 15 min at 25 °C before being quenched in 0.125 M glycine for 5 min. The rinsed cells were then lysed in ChIP lysis buffer (50 mM Tris-HCl pH 8.0, 1% sodium dodecyl sulfate, 10 mM EDTA) and sonicated on ice with a Fisher Sonic Dismembrator 60 (output 4.5, 20 s pulse repeated 6 times on ice with 40 s between each sonication). The supernatant containing sonicated chromatin was pre-cleared by the addition of magnetic Protein G dynabeads (Thermo Fisher) that had been pretreated with denatured salmon sperm DNA (Invitrogen). The magnetic beads were removed, and the sonicated chromatin was incubated overnight at 4 °C using 2.5 µg of the indicated antibody in ChIP dilution buffer (16.7 mM Tris-HCL pH 8.0, 1% Triton X-100, 0.01% SDS, 150 mM NaCl, 1.2 mM EDTA). The sonicated chromatin (5%) was stored as input DNA without further treatment until the reverse crosslinking step.

Protein G dynabeads were then added to the chromatin-antibody mixture, incubated for 2 h at 4 °C and then washed 3 times with ChIP LiCl buffer (10 mM Tris-HCL pH 8.0, 1% NP-40, 250 mM LiCl, 1 mM EDTA, 1% Na deoxycholate) and twice with TE buffer (10 mM Tris-HCL pH 8.0, 1 mM EDTA). Protein-DNA complexes were eluted from the beads with an elution buffer (0.1 M NaHCO3, 1% SDS), de-crosslinked by incubating at 65 °C for 16 h and at 95 °C for 15 min, then digested by adding 50 μg proteinase K and incubating at 50 °C for 3 h. DNA was extracted using a DNA Miniprep Plus kit (Zymo), digested with DpnI (NEB) to remove any plasmid contamination, then used for qPCR analysis using primers that amplify U5-R on the HIV-1 promoter (FP: 5′- CTCTCTGGTTAGACCAGATC-3′, RP: 5′-GCTAGAGATTTTCCACACTG-3′). ChIP data are expressed as a percentage of input DNA.

### Rescue of ΔNSMCE2 knockout cells

A lentiviral vector expressing a FLAG-tagged NSMCE2 protein that is resistant to cutting by the sgRNA expressed in the CEM-SS ΔNSMCE2 knockout cells was created by mutating the NSMCE2 sequence from an expression plasmid (OHu31586, GenScript) via overlap extension PCR to introduce synonymous T-C and C-T mutations into the sgRNA target sequence (GTATCAACTCTGGTATGGAC to GcATtAACTCTGGTATGGAC). This mutant FLAG-NSMCE2 PCR product was then cloned into the pLCE lentiviral vector using NheI and XhoI restriction sites.

Similarly, the NSMCE2ΔSUMO mutant was created using overlap extension PCR to introduce the C185S and H187Q amino acid substitutions into the RING domain necessary for E3 SUMO ligase function^25^.

Lentiviruses were created from pLCE (control), pLCE FLAG-NSMCE2 and pLCE FLAG-NSMCE2ΔSUMO, which were then used to transduce CEM-SS or CEM-SS ΔNSMCE2 cells. These cells were infected with IN+/IN− NL-NLuc or IN− NL-GFP at 0.3 MOI and the cells were collected at 2 dpi for the respective NLuc assays (NL-NLuc), or for flow cytometry and ChIP–qPCR (NL-GFP). Expression of these NSMCE2 constructs was validated by western blot.

### E3 SUMO ligase inhibition kinetics

TAK-981 (MedChemExpress) is a global inhibitor of SUMOylation^26^ and was reconstituted in DMSO to a 5 mM stock. TAK-981 was added to CEM-SS Cas9 and ΔSMC5 cells infected with NL-NLuc at 0, 5, 15, 30, 75, 150, 500 and 1,000 nM concentrations, and the cells were collected for an NLuc assay after 2 dpi.

To assay the effects of TAK-981 on viral RNA expression in infected cells, CEM-SS cells were infected with IN+/IN− NL-NLuc in the presence or absence of 150 nM TAK-981. RNA was extracted at 2 dpi with TRIzol (Thermo Fisher) and DNAse I treated for 2 h. cDNA was then made using the High Capacity cDNA Reverse Transcription kit (Applied Biosystems). The levels of unspliced HIV-1 RNA gag or the spliced viral RNA from donor 1 acceptor 1 (A1D1) and splice donor 7 acceptor 4 (A4D7) were quantified by qPCR using the primers for gag (FP: 5′-GCGAGAGCGTCGGTATTAAGCG-3′, RP: 5′-AATCGTTCTAGCTCCCTGCTTGC-3′), A1D1 (FP: 5′-GATCTCTCGACGCAGGACTC-3′, RP: 5′-TGGTCCTTTCCAAACTGGAT-3′) and A4D7 (FP: 5′- CAAGCTTCTCTATCAAAGCAACC -3′, RP: 5’- AATCGAATGGATCTGTCTCTGTC -3′)^55^.

To understand the kinetics of inhibiting SUMOylation on viral gene expression and epigenetic modifications in infected cells, 150 nM TAK-981 was added to CEM-SS Cas9 and ΔSMC5 cells at the time of infection, or at 16, 19, 21, 24, 36 and 48 hpi. Cells were infected with NL-NLuc Δenv and collected at 72 hpi for either NLuc assays or ChIP–qPCR using anti-SUMO2/3, H3Ac or H3K9me3 antibodies and quantifying the HIV-1 promoter using primers that amplify the U5-R region (FP: 5’- CTCTCTGGTTAGACCAGATC-3′, RP: 5’-GCTAGAGATTTTCCACACTG-3′). ChIP data are expressed as a percentage of input DNA.

### HIV-1 latency quantification

#### CEM-SS latency assay

WT or ΔSMC5 CEM-SS cells were infected with the IN+ NL-GFPΔEnv virus at an MOI of ~0.1 in the presence or absence of 150 nM TAK-981. Nevirapine was added to the cells at 2 dpi to inhibit any late infection events, and at 3 dpi the cells were washed and resuspended in 2% BSA in PBS for sorting on a BSL-3 contained FACS Aria II (BD Biosciences) to isolate GFP− cells. These cells were allowed to recover in growth media for 6 d (9 dpi) before treatment with DMSO, 150 nM TAK-981, 80 nM PMA or 1 ng ml^−1^ TNF-α. Cells were collected the next day (10 dpi) to analyse GFP expression by flow cytometry.

### CEM-SS delayed integration latency assay

WT or ΔSMC5 CEM-SS cells were infected with the IN+ NL-GFPΔEnv virus in the presence or absence of 500 nM raltegravir. At 2 dpi, all cells were washed three times with PBS, replated in RPMI with nevirapine and cultured for an additional 3 d. At 5 dpi, GFP− cells were sorted, allowed to recover in growth media for 6 d, treated with DMSO, PMA or TNF-α (11 dpi) and analysed for flow cytometry the next day (12 dpi).

Both CEM-SS control and ΔSMC5 cells were infected with a normalized p24 amount that was previously titred to give 10% GFP+ cells in CEM-SS cells +Ral (~0.45 MOI) and −Ral (0.1 MOI) at 5 dpi.

#### Primary activated CD4+ T cell latency assay

Activated primary T cells isolated from PBMCs were infected at a low MOI of ~0.05 with an IN+ NL-DC reporter virus that expresses eGFP off the HIV-1 LTR and mCherry from an internal EF1-α promoter. Infections were carried out in the presence and absence of 150 nM TAK-981 added at infection, or with 80 nM PMA known to reverse epigenetic silencing of HIV-1 in latent cells^56^ or 150 nM TAK-981 added 24 h before collection. Cells were collected for flow cytometry at 7 dpi in 2% BSA in PBS to measure eGFP and mCherry fluorescence. The CD4+ T cell population was separated from dead cells and cellular debris by gating on forward and side-scatter analyses. The high enrichment by the CD4 Positive Isolation kit (Invitrogen) also allowed us to identify the enriched CD4+ T cell population and gating was done to exclude the few possible CD4+ monocytes that might be present. Flow cytometry data were then analysed using FlowJo v10.6.2.

DNA was also extracted from these cells at 2, 3, 5 and 7 dpi, incubated with DpnI to remove residual HIV-1 plasmid contaminants, then the amounts of unintegrated 2LTR HIV-1 circular DNA quantified by qPCR as described in a previous section.

### Statistical analysis

Sample size and *P* values are indicated in the text or figure legends. Error bars in the experiments represent standard deviations of the mean from independent experiments. Statistical analyses were performed using GraphPad Prism or reported by the indicated computational tools used in the analysis of the CRISPR knockout screen. Information about statistical methods is specified in the figure legends.

### Materials availability

All unique materials generated in this study will be made available upon reasonable request to the lead contact and will also be made available through the NIH AIDS Reagent programme.

### Reporting summary

Further information on research design is available in the Nature Research Reporting Summary linked to this article.

### Supplementary information

Supplementary Information Supplementary Information.

Reporting Summary

## Data Availability

The data that support the findings of this study are available from the corresponding author upon request. Sequencing data generated in this study are available from the NCBI Sequence Read Archive with the dataset identifier SRR18245559 (CRISPR Knockout Screen). Source data are provided with this paper.

## Source data

Source Data Fig. 1 Statistical source data.
Source Data Fig. 2 Statistical source data.
Source Data Fig. 2 Unprocessed western blots.
Source Data Fig. 3 Statistical source data.
Source Data Fig. 4 Statistical source data.
Source Data Fig. 5 Statistical source data.
Source Data Extended Data Fig. 1 Statistical source data.
Source Data Extended Data Fig. 2 Unprocessed western blots.
Source Data Extended Data Fig. 3 Statistical source data.
Source Data Extended Data Fig. 4 Statistical source data.
Source Data Extended Data Fig. 5 Statistical source data.
Source Data Extended Data Fig. 6 Statistical source data.
Source Data Extended Data Fig. 7 Statistical source data.
Source Data Extended Data Fig. 8 Statistical source data.
