# Supplementary material for: Epigenetic silencing by the SMC5/6 complex mediates HIV-1 latency
Source: Nat Microbiol. 2022 Nov 14;7(12):2101–13. doi: 10.1038/s41564-022-01264-z (PMC9712108; doi:10.1038/s41564-022-01264-z)
Supplement: Supplementary file 1 — Supplementary Information. [file 41564_2022_1264_MOESM1_ESM.pdf]

nature microbiology

Article https://doi.org/10.1038/s41564-022-01264-z

Epigenetic silencing by the SMC5/6 complex mediates HIV-1 latency

In the format provided by the authors and unedited

Supplementary Information

Crispr KO Clone	Sequence 1	Sequence 2	Sequence 3
ΔSMC5.1 c4	1bp deletion<br>exon 8	8bp deletion<br>exon 8	11bp deletion<br>exon 8
ΔSMC5.1 c17	2bp deletion<br>exon 8	11bp deletion<br>exon 8	18bp deletion into intron 7 exon 8 junction
ΔSMC6.2 c10	2bp deletion<br>exon 6	7bp deletion<br>exon 6	17bp deletion<br>exon 6
ΔSMC6.2 c11	1bp deletion<br>exon 6	5bp deletion<br>exon 6	10bp deletion<br>exon 6
ΔNSMCE2.3 c9	7bp deletion<br>exon 3	16bp deletion<br>exon 3	NA
ΔNSMCE4.4 c2	5bp deletion<br>exon 6	14bp deletion<br>exon 6	19bp deletion<br>exon 6
ΔNSMCE4.4 c12	2bp deletion<br>exon 6	7bp deletion<br>exon 6	28bp deletion<br>exon 6
ΔSLF1.2 c14	1bp insertion<br>exon 4	515bp deletion<br>exon 4 - intron 4	NA

ΔSLF1.4 c4	7bp deletion<br><br>exon 10	1bp insertion<br><br>exon 10	NA
ΔSLF2.1 c1	248bp insertion<br><br>exon 3	11bp deletion<br><br>exon 3	NA
ΔSLF2.2 c4	1bp insertion<br><br>exon 6	2bp deletion<br><br>exon 6	NA

Supplementary Table S1: Description of the mutations introduced into the genes encoding the indicated SMC5/6 components by gene editing using CRISPR/Cas and detected by Sanger sequencing. All mutations are predicted to introduce frame shifts.
