# Supplementary material for: Epigenetic silencing by the SMC5/6 complex mediates HIV-1 latency
Source: Nat Microbiol. 2022 Nov 14;7(12):2101–13. doi: 10.1038/s41564-022-01264-z (PMC9712108; doi:10.1038/s41564-022-01264-z)
Supplement: Supplementary file 2 — Reporting Summary [file 41564_2022_1264_MOESM2_ESM.pdf]

nature portfolio

Corresponding author(s): Bryan R. Cullen

Last updated by author(s): Oct 4, 2022

Reporting Summary

Nature Portfolio wishes to improve the reproducibility of the work that we publish. This form provides structure for consistency and transparency in reporting. For further information on Nature Portfolio policies, see our Editorial Policies and the Editorial Policy Checklist.

Statistics

For all statistical analyses, confirm that the following items are present in the figure legend, table legend, main text, or Methods section.

n/a	Confirmed	
☐	☒	The exact sample size (*n*) for each experimental group/condition, given as a discrete number and unit of measurement
☐	☒	A statement on whether measurements were taken from distinct samples or whether the same sample was measured repeatedly
☐	☒	The statistical test(s) used AND whether they are one- or two-sided<br>*Only common tests should be described solely by name; describe more complex techniques in the Methods section.*
☒	☐	A description of all covariates tested
☐	☒	A description of any assumptions or corrections, such as tests of normality and adjustment for multiple comparisons
☐	☒	A full description of the statistical parameters including central tendency (e.g. means) or other basic estimates (e.g. regression coefficient) AND variation (e.g. standard deviation) or associated estimates of uncertainty (e.g. confidence intervals)
☐	☒	For null hypothesis testing, the test statistic (e.g. *F*, *t*, *r*) with confidence intervals, effect sizes, degrees of freedom and *P* value noted<br>*Give P values as exact values whenever suitable.*
☒	☐	For Bayesian analysis, information on the choice of priors and Markov chain Monte Carlo settings
☒	☐	For hierarchical and complex designs, identification of the appropriate level for tests and full reporting of outcomes
☒	☐	Estimates of effect sizes (e.g. Cohen's *d*, Pearson's *r*), indicating how they were calculated

Our web collection on statistics for biologists contains articles on many of the points above.

Software and code

Policy information about availability of computer code

Data collection	Flow cytometry data was collected on a FACS Aria II (for cell sorting), or Fortessa X20 running BD FACSDIVA.
Data analysis	Flow cytometry was analyzed with FlowJo v10.6.2<br><br>Crispr Screens were analyzed with MAGECK-VISPR 0.5.6<br><br>Statistical analysis<br>Data were analyzed with GraphPad software v.9.4

For manuscripts utilizing custom algorithms or software that are central to the research but not yet described in published literature, software must be made available to editors and reviewers. We strongly encourage code deposition in a community repository (e.g. GitHub). See the Nature Portfolio guidelines for submitting code & software for further information.

Data

Policy information about availability of data

All manuscripts must include a data availability statement. This statement should provide the following information, where applicable:

- Accession codes, unique identifiers, or web links for publicly available datasets
- A description of any restrictions on data availability
- For clinical datasets or third party data, please ensure that the statement adheres to our policy

Sequencing data generated in this study is available from the NCBI Sequence Read Archive with the dataset identifier SRR18245559 (Crispr Knockout Screen).

Human research participants

Policy information about studies involving human research participants and Sex and Gender in Research.

Reporting on sex and gender	This information has not been collected. We have processed no human research participants.
Population characteristics	See above.
Recruitment	See above.
Ethics oversight	Study does not involve human research and is not subject to IRB ethics oversight. Whole blood was bought from a third party (Gulf Coast Regional Blood Center) with no interaction or intervention with the donor. Blood was de-identified prior to purchasing, and no identifiable private information was generated during the course of research.

Note that full information on the approval of the study protocol must also be provided in the manuscript.

Field-specific reporting

Please select the one below that is the best fit for your research. If you are not sure, read the appropriate sections before making your selection.

☒ Life sciences ☐ Behavioural & social sciences ☐ Ecological, evolutionary & environmental sciences

For a reference copy of the document with all sections, see nature.com/documents/nr-reporting-summary-flat.pdf

Life sciences study design

All studies must disclose on these points even when the disclosure is negative.

Sample size	Experiments were performed at least in triplicate. A minimum of n=3 was chosen as a sample size to generate robust and reliable results on the cell lines based on similar studies in the field.
Data exclusions	No data was excluded.
Replication	Most of the experiments were repeated a minimum of three times, not finding any reproducibility problems
Randomization	Randomization is not relevant to this study. The experiments in this study compare different mammalian cells with defined KO mutations, genotypes, or lentiviral integrations. A selection bias does not affect the results in these studies. Samples and their appropriate controls were processed at the same time.
Blinding	Blinding was not carried out in this study. The relevant controls were processed at the same time as the samples and read in an unbiased manner.

Reporting for specific materials, systems and methods

We require information from authors about some types of materials, experimental systems and methods used in many studies. Here, indicate whether each material, system or method listed is relevant to your study. If you are not sure if a list item applies to your research, read the appropriate section before selecting a response.

Materials & experimental systems

n/a	Involved in the study	
☐	☒	Antibodies
☐	☒	Eukaryotic cell lines
☒	☐	Palaeontology and archaeology
☒	☐	Animals and other organisms
☒	☐	Clinical data
☒	☐	Dual use research of concern

Methods

n/a	Involved in the study	
☒	☐	ChIP-seq
☐	☒	Flow cytometry
☒	☐	MRI-based neuroimaging

Antibodies

Antibodies used

Mouse anti-CD28/CD49d BD Biosciences 347690; RRID:AB_647457
Rabbit anti-SMC5 Invitrogen PA5-115931; RRID:AB_2900565
Rabbit anti-SMC6 Invitrogen PA5-80042; RRID:AB_2747157
Rabbit anti-NSMCE2 Proteintech 13627-1-AP; RRID:AB_10637854
Rabbit anti-NSMCE4 GeneTex GTX121270; RRID:AB_11169701
Rabbit anti-SLF1 Abgent AP5407a; RRID:AB_10816722
Rabbit anti-SLF2 Abcam ab122480; RRID:AB_11129755
Rabbit anti-mouse IgG Abcam ab46540; RRID:AB_2614925
Rabbit anti-H3Ac EMD Millipore 06-599; RRID:AB_2115283
Rabbit anti-H3K4me3 Cell Signaling 9751S; RRID:AB_2616028
Rabbit anti-H3K9me3 Abcam ab8898; RRID:AB_306848
Rabbit anti-H3K27me3 Cell Signaling 9733S; RRID:AB_2616029
Mouse anti-BActin Proteintech 66009-1-Ig; RRID:AB_2687938
Anti-Rabbit HRP Sigma cat#A6154; RRID:AB_258284
Anti-Mouse HRP Sigma cat#A9044, RRID:AB258431
Rabbit anti-SUMO2/3 Abcam ab3742; RRID:AB_304041
Mouse anti-FLAG Sigma F3165; RRID:AB_259529

Validation

All these antibodies are commercially available with validation data available on the manufacturer's websites.
Mouse anti-CD28/CD49d antibodies are characterized and validated by the manufacturer for use in activating human T cells.
Rabbit antibodies against SMC5, SMC6, NSMCE2, NSMCE4, SLF1, SLF2, and beta actin have all been validated for western blotting and are shown to be able to specifically detect the proteins isolated from human cells.
The anti-mouse and -rabbit HRP antibodies are specifically developed for western blotting.
H3Ac,H3K4me3, H3K9me3, H3K27me3 antibodies are advertised and validated on the manufacturers website for ChIP in human cell lysates. In addition these antibodies have been characterized to standard by the ENCODE project.
FLAG antibodies used have been well validated for various applications, including western blotting and ChIP.
SUMO2/3 antibodies are validated by the manufacturer for western blot on human samples, and have been successfully used in ChIP-seq experiments (https://doi.org/10.1186/1471-2164-14-824).

Eukaryotic cell lines

Policy information about cell lines and Sex and Gender in Research

Cell line source(s)

293T cells were from the ATCC
CEM-SS cells were from AIDS Reagent

Authentication

No further authentication.

Mycoplasma contamination

Cell lines were routinely tested for mycoplasma contamination, and we have obtained negative results throughout the duration of the study.

Commonly misidentified lines
(See ICLAC register)

No commonly misidentified cell lines were used in this study

Flow Cytometry

Plots

Confirm that:

☒ The axis labels state the marker and fluorochrome used (e.g. CD4-FITC).

☒ The axis scales are clearly visible. Include numbers along axes only for bottom left plot of group (a 'group' is an analysis of identical markers).

☒ All plots are contour plots with outliers or pseudocolor plots.

☒ A numerical value for number of cells or percentage (with statistics) is provided.

Methodology

Sample preparation	Peripheral Blood Mononuclear Cells (PBMC) were isolated from whole blood by density gradient centrifugation over Histopaque (Sigma), and CD4+ cells were then isolated using CD4 Positive Isolation kit (Invitrogen). Isolated CD4+ cells were then activated by incubation with antibodies against CD3/CD28 /CD49d (BD Biosciences) and 5ug/ml phytohemagglutinin (PHA) in RPMI supplemented with 10% FBS and 10% IL-2.<br><br>CEM-SS and its derivatives were cultured in RPMI with 10% FBS.
Instrument	FACS Aria II (for cell sorting), or Fortessa X20 running BD FACSDIVA.
Software	FlowJo v10.6.2
Cell population abundance	Data analyses were performed using at least 10,000 live T cells in each anaysis.
Gating strategy	For all the experiments, live T cells were gated on forward vs. side scatter plot based on their size and granularity. Single cells were gated on a forward scatter height vs. forward scatter area plot. The gates for GFP+ and/or GFP- cells are defined according to the uninfected controls and are apparent in the figures.

☒ Tick this box to confirm that a figure exemplifying the gating strategy is provided in the Supplementary Information.
