# Supplementary material for: Epigenetic silencing by the SMC5/6 complex mediates HIV-1 latency
Source: Nat Microbiol. 2022 Nov 14;7(12):2101–13. doi: 10.1038/s41564-022-01264-z (PMC9712108; doi:10.1038/s41564-022-01264-z)
Supplement: Source Data Extended Data Fig. 2 — Unprocessed western blots. [file 41564_2022_1264_MOESM10_ESM.pdf]

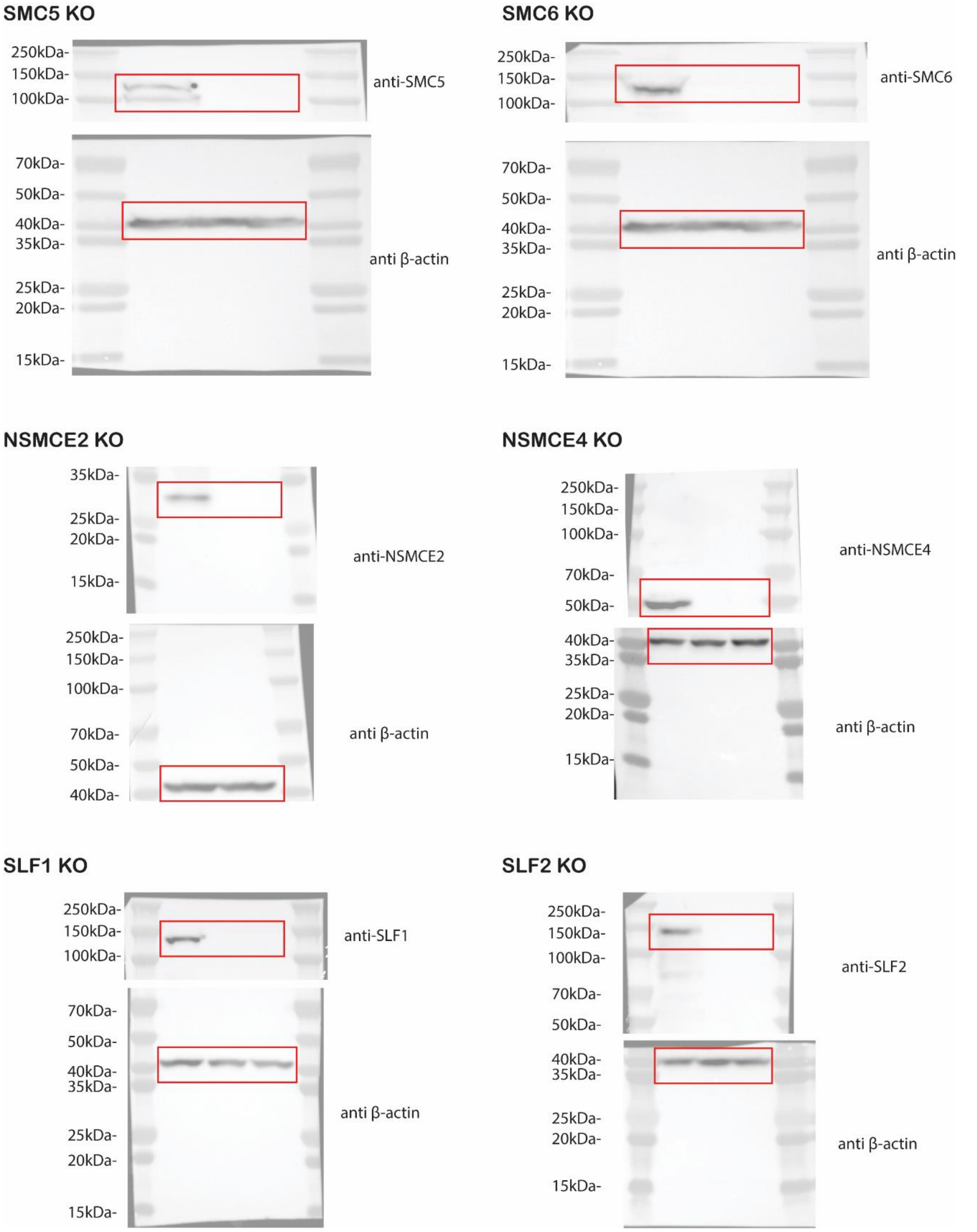

Immunoblots for Extended Fig. 2: Uncropped membranes are shown, with the rectangles indicating cropped regions. Protein ladder sizes are indicated.
